# Complement Membrane Attack Complexes Disrupt Proteostasis to Function as Intracellular Alarmins

**DOI:** 10.21203/rs.3.rs-4504419/v1

**Published:** 2024-06-19

**Authors:** Dan Jane-wit, Guiyu Song, Liying He, Quan Jiang, Mahsa Barkestani, Shaoxun Wang, Qianxun Wang, Pengwei Ren, Matthew Fan, Justin Johnson, Clancy Mullan

**Affiliations:** Yale University; Yale School of Medicine; Yale University; Yale University; Yale University; Yale University; Yale University; Yale University School of Medicine; Yale University School of Medicine; Yale University School of Medicine; Yale School of Medicine

**Keywords:** membrane attack complex, alarmin, complement, endothelial cell, aggrephagy, macroautophagy, ZFYVE21, RNF34, Rab5, endosome

## Abstract

Internalized pools of membrane attack complexes (MACs) promote NF-kB and dysregulated tissue inflammation. Here, we show that C9, a MAC-associated protein, promotes loss of proteostasis to become intrinsically immunogenic. Surface-bound C9 is internalized into Rab5 + endosomes whose intraluminal acidification promotes C9 aggregates. A region within the MACPF/CDC domain of C9 stimulates aggrephagy to induce NF-kB, inflammatory genes, and EC activation. This process requires ZFYVE21, a Rab5 effector, which links LC3A/B on aggresome membranes to RNF34-P62 complexes to mediate C9 aggrephagy. C9 aggregates form in human tissues, C9-associated signaling responses occur in three mouse models, and ZFYVE21 stabilizes RNF34 to promote C9 aggrephagy *in vivo*. Gene-deficient mice lacking ZFYVE21 in ECs showed reduced MAC-induced tissue injury in a skin model of chronic rejection. While classically defined as cytotoxic effectors, MACs may impair proteostasis, forming aggregates that behave as intracellular alarmins.

## INTRODUCTION

Membrane attack complexes (MACs) are immune mediators promoting tissue injury^1^ that have been therapeutically targeted in ~ 40 inflammatory conditions.^2^ MACs share structural and functional properties with perforin and efficiently promote osmolysis of anucleated red blood cells^3^ and xenogeneic pathogens.^4,5^ Extrapolating from these effects, a majority of studies have attributed pro-inflammatory effects of MACs to their abilities to cause cell death.^1,6–8^

Complement proteins inclusive of MACs show diagnostic,^9^ prognostic,^10^ and therapeutic value^11–13^ in chronic antibody-mediated rejection (CABMR). In CABMR, MACs principally assemble on autologous, nucleated endothelial cells (ECs), and this is associated with dysregulated inflammation but not diffuse EC death.^14–16^ A similar process occurs in connective tissue disorders,^17^ autoimmune vasculitis,^18^ and viral infection including SARS-CoV-2,^19,20^ suggesting a generalizable form of immunogenicity linked to MACs that is separable from their cytolytic effects.

We have used ‘high’ panel reactive antibody (PRA) sera,^21^ termed ‘PRA,’ to model non-cytolytic effects of MACs on ECs, principal targets of MACs during CABMR.^22^ Recapitulating clinical observations, PRA-induced inflammatory signals occurred in ECs without causing cell death.^22^ Using PRA, we unexpectedly observed that intracellular MACs were salient activators of NF-kB. Blockade of clathrin-mediated endocytosis (CME),^23^ Rab5 activity,^24–26^ or Rab5 effectors^24,26–27^ abrogated NF-kB, indicating that intracellular but not extracellular or surface-bound MACs primarily elicited EC activation.

A single MAC pore contains ~ 20–25 C’ proteins with a molecular weight of ~ 1,600 kD. This *de novo* protein burden on ECs is anticipated to significantly challenge proteostasis, particularly during chronic inflammatory states like CABMR where MACs may become persistently assembled and internalized. C9 comprises the majority of MACs with 13–18 molecules per MAC; and ~ 7–8% of C9 consists of intrinsically disordered regions (IDRs, https://mobidb.bio.unipd.it, http://biocomp.chem.uw.edu.pl/A3D2), suggesting susceptibility to aggregate formation. Alarmins are endogenous proteins that are basally non-immunogenic but become pro-inflammatory after losing their homeostatic compartmentalization^28^ or after becoming pathologically modified as is the case for amyloidogenic proteins.^29^ Based on the spatially restricted responses of MACs and the aggregate-prone features of its majority component, C9, we tested the hypothesis that intracellular C9 impairs proteostasis in ECs, generating aggregates that operationally behave as alarmins.

## RESULTS

### Protein Aggregates and EC Activation in CABMR Biopsies.

**Figure 1:**
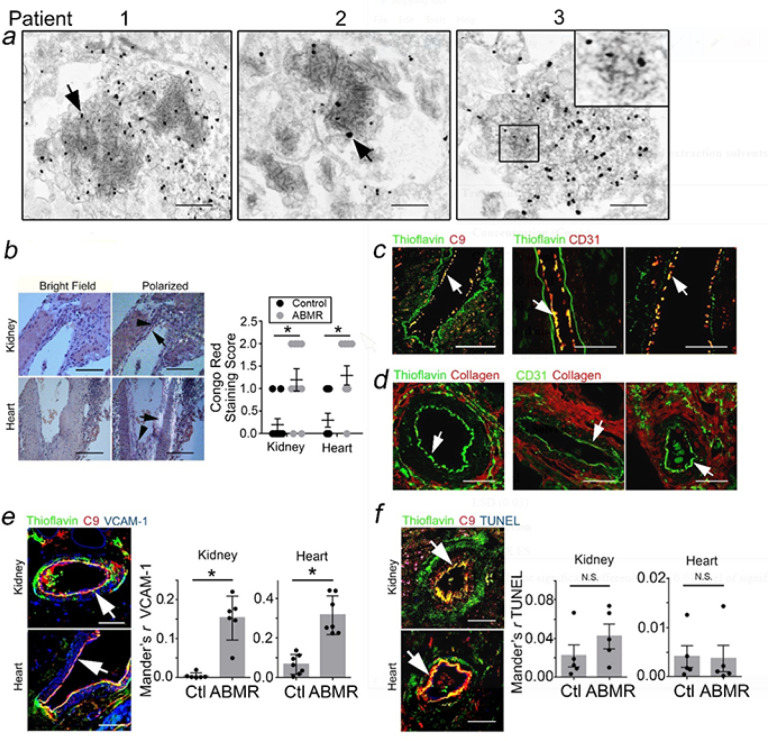
Protein Aggregates and EC Activation in CABMR Biopsies. C9 immune-EM of adluminal cells in arterioles of CABMR kidney biopsies. Scale Bar = 5 μm (*a*, n=8). Congo Red staining was quantified in 4–8 hpfs in kidney and heart CABMR biopsies. Scale Bar = 200 μm (*b*). Kidney and heart biopsies were analyzed by I.F. for thioflavin, C9, CD31, and collagen (*c,d*). Colocalization of VCAM-1 (*e*) and TUNEL (*f*) with Thioflavin+C9+ adluminal regions were quantified in 5 hpfs. Scale Bar = 5μm (*a*), 200μm (*b-d*), and 100μm (*e,f*). *p<0.05 using Student’s t-test (*b,d,e*).

We initially surveyed CABMR biopsies, excluding patients with confounding conditions characterized by protein aggregates including primary amyloidosis and post-transplant lymphoproliferative disorder (PTLD, Supplementary Table 1).^30^ In immune-EM, we observed that certain adluminal cells contained filamentous inclusions enriched for C9 which appeared to be contained within intracellular vesicles (Fig. 1a). We did not detect filamentous inclusions in glomerular ECs or in control tissues from transplant patients undergoing routine surveillance biopsies.

Congo Red and thioflavin clinically identify protein aggregates in tissues, but these dyes also reportedly fluoresce in the presence of collagen fibrils.^31,32^ We indeed observed dye fluorescence in laminations in arterioles with allograft vasculopathy (AV, Supplementary Fig. 1a), sites well known to contain collagen,^9^ as well as morphologically fibrosed interstitial regions (Supplementary Fig. 1b). The concurrent presence of fibrosis precluded evaluation of putative protein aggregates at these sites. However, in 3 of 8 patients we surprisingly observed congophilic staining in adluminal cells lacking AV (Fig. 1b). These adluminal cells costained for C9 and thioflavin (arrows, Fig. 1c) but not collagen (arrows, Fig. 1d). C9 + Thioflavin + vessels moreover expressed VCAM-1 (Fig. 1e) at ~ 3- to 10-fold higher levels than TUNEL (Fig. 1f).

### The C9 Component of MACs Forms Aggregates in ECs.

**Figure 2:**
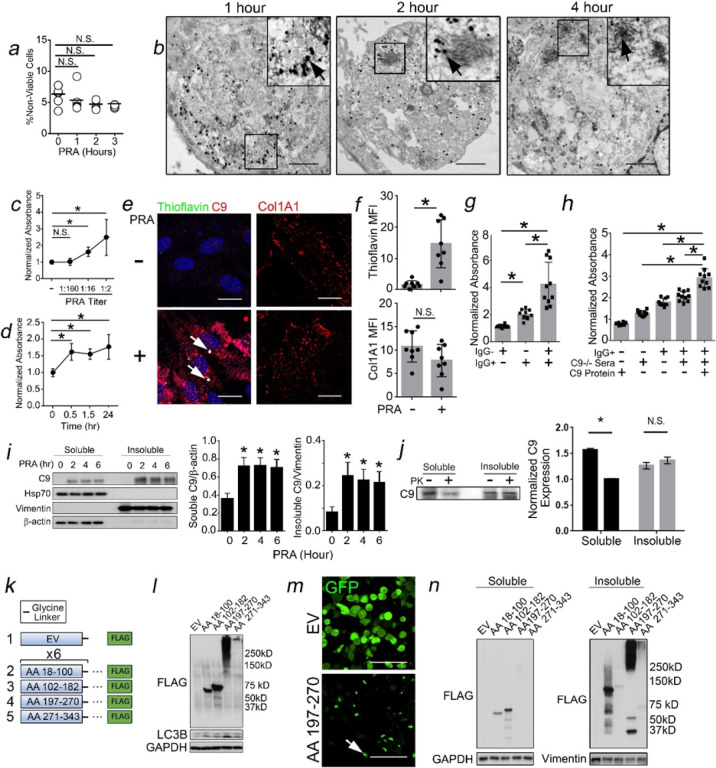
The C9 Component of MACs Forms Aggregates in Human ECs. HUVECs pre-treated with IFN-g (50ng/mL) for 48 hours were treated with PRA for various times prior to viability dye staining and analysis by FACS (*a*). Immune-EM for C9 (arrows) in HUVECs over time following PRA treatment (*b*). HUVECs were treated with PRA at various dilutions, and thioflavin fluorescence was assessed at 2 hours (*c*). Thioflavin fluorescence was assessed at various times following treatment with PRA at 1:4 dilution (*d*). Punctae containing thioflavin and C9 (*e*) but not collagen (*f*) were increased in PRA-treated HUVECs by I.F. at 2 hours. PRA sera were fractionated into IgG- and IgG+ fractions and added to HUVECs for 2 hours prior to assessing thioflavin fluorescence (*g*). IgG+ fractions of PRA sera were added to HUVECs with and without C9-deficient reference sera and C9 protein (5 mg/mL) for 2 hours prior to assessing thioflavin fluorescence (*h*). HUVECs were treated with PRA for various times, and soluble and insoluble lysate fractions were analyzed by Western blot (*i*). Human C9 (0.125 mg/mL) was incubated at room temperature with proteinase K (12.5 μg/ml mg/mL) for 30 minutes prior to Western blot analysis (*j*). AA197–270 cells (*k*) showed high molecular weight (*l*) punctate (*m*) aggregates that became SDS-insoluble (*n*). Experiments repeated ≥ 3 times using different HUVEC donors. Scale Bar = 5mm (*b*), 15mm (*e*), 50mm (*m*). *p<0.05 using one-way ANOVA (*i*) or two-way ANOVA (*a,c-d,g-h*) with Tukey’s post-hoc correction or Student’s t-test (*f,j*).

We used PRA-treated HUVECs to mechanistically analyze patient-level findings. As previously observed,^22^ PRA-treated HUVECs did not show increased cell death (Fig. 2a). In kinetic immune-EM studies, PRA caused C9 to colocalize within large, perinuclear structures (Fig. 2b) developing filamentous inclusions (Fig. 2b). Contemporaneously, thioflavin fluorescence increased in both a dose- and time-dependent manner (Fig. 2c,d). Under I.F., thioflavin showed punctate staining colocalizing with C9 in large, perinuclear vesicles (Fig. 2e). Thioflavin but not type I collagen fluorescence increased with PRA (Fig. 2e,f), excluding confounding collagen staining as a cause for increased thioflavin signals. These results recapitulated ultrastructural and histologic findings in CABMR.

Following treatment, PRA generates alloAb, anaphylatoxins, and MACs. To identify culprit mediator(s) causing thioflavin fluorescence, we performed sera fractionation and recombination studies. We separated PRA sera into its IgG + and IgG- fractions and found that while these fractions showed only minimal effects individually, combining the IgG- and IgG + fractions significantly potentiated thioflavin fluorescence (Fig. 2g). This ruled out IgG or contaminant(s) as a principal cause for increased thioflavin staining and suggested a role for C’ activity.

To test C9 aggregation, we treated HUVECs with the IgG + fraction of PRA combined with C9-deficient reference sera. This permitted IgG binding, anaphylatoxin formation, and oligomerization of MAC proteins to form C5b-8 which has pore-forming capabilities^33^ but lacks C9. IgG-induced C’ activation with C9-deficient sera minimally increased thioflavin fluorescence compared to the IgG + fraction alone (Fig. 2h, lane 3 *vs* lane 4). Addition of C9, which alone showed no effects (lane 1) significantly rescued thioflavin staining (lane 4 *vs* lane 5). We separated PRA-treated HUVECs into soluble and insoluble fractions, and detected increased C9 within the SDS-insoluble pellet (Fig. 2i) that, unlike pools of C9 within SDS-soluble supernatants, became resistant to mild proteinase K (PK) digestion, together indicating generation of insoluble C9 (Fig. 2j).

To further verify C9 aggregates, we tandemly expressed elements within the TSP, LDLRA, and MACPF domains of C9 that form b-sheets (www.uniprot.org,prosite.expasy.org/scanprosite/), a conformation frequently adopted in protein aggregates. Each element, ranging from ~ 6–9 kD, was tandemly expressed intracellularly and separated by a flexible glycine linker to allow antiparallel b-sheet stacking which occurs upon native assembly of MACs (Fig. 2k). We linked these elements to FLAG as this tag is less prone to aggregation relative to GFP, RFP, and luciferase reporters.

Of elements tested, AA197–270, an element within the MACPF domain containing the majority of predicted IDRs, strongly induced high molecular weight aggregates in HEK293 cells (Fig. 2l). AA197–270 colocalized within large perinuclear punctae, phenocopying distributions of native C9 aggregates in PRA-treated HUVECs (Fig. 2m). In fractionation studies, AA197–270 aggregates exclusively appeared within the insoluble pellet (Fig. 2n). Original and uncropped Western blot films for Fig. 2 are shown in Supplementary Fig. 3. C9 may form aggregates.

### Intracellular C9 Form Aggregates Within the Endolysosomal Pathway.

**Figure 3:**
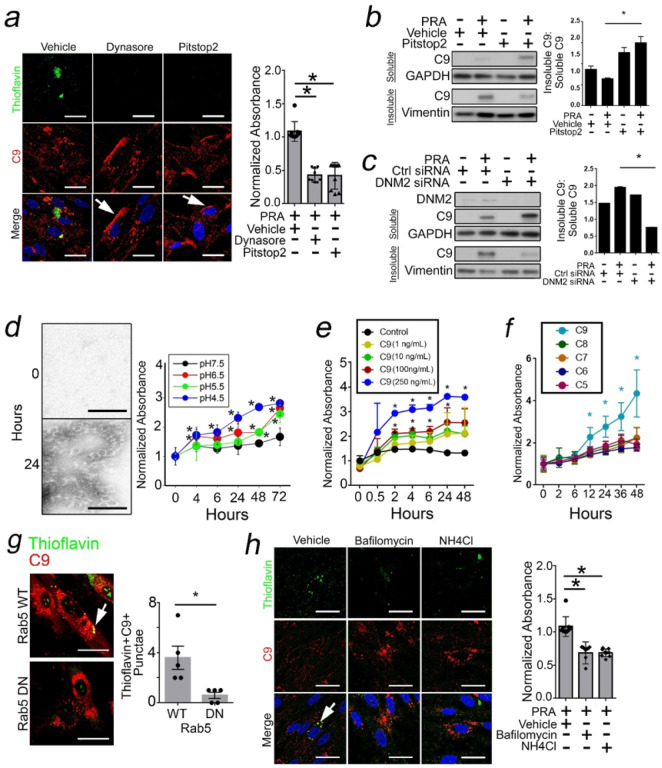
Intracellular C9 Form Aggregates Within the Endolysosomal Pathway. HUVECs were pre-treated with vehicle, Dynasore (80 mg/mL), or Pitstop2 (30 mg/mL) for 30 minutes prior to addition of PRA for 4 hours prior to assessing thioflavin fluorescence (*a*). PRA-treated HUVECs were exposed to vehicle or Pitstop2, and soluble and insoluble lysates were assessed for C9 by Western blot (*b*). HUVECs transfected with control or DNM2 siRNA were treated with PRA, and soluble and insoluble lysates were assessed for C9 (*c*). Human C9 protein (0.125 mg/mL) was resuspended in buffers of varying pH, and thioflavin fluorescence was assessed over time at 37°C (*d*). Human C9 protein at concentrations indicated were incubated at 37°C and thioflavin fluorescence was assessed over time (*e*). Human complement proteins at 0.125 mg/mL were incubated at times indicated at pH 4.5 at 37°C (*f*). HUVECs were transduced with Rab5 WT and Rab5 DN constructs and treated with PRA for 2 hours prior to I.F. analysis (*g*). HUVECs were exposed to vehicle, bafilomycin (100 nM), or NH_4_Cl (10 mM) for 2 hours, PRA was added, and thioflavin fluorescence was assessed 4 hours later (*h*). Experiments repeated ≥ 3 times using different HUVEC donors. Scale Bar = 30mm (*a,g,h*), 20mm (*d*). *p<0.05 using one-way ANOVA (*h*) or two-way (a-c) ANOVA with Tukey’s post-hoc correction or Student’s t-test (g).

We asked whether C9 aggregates formed at the cell surface and/or intracellularly. We previously found that MAC internalization required CME.^23^ Exploiting this, we pre-treated HUVECs with CME inhibitors, Dynasore and PitStop2, and tested effects on C9 aggregates. Both CME inhibitors increased C9 levels at the cell surface (Fig. 3a, arrows), and this significantly reduced thioflavin fluorescence in PRA-treated HUVECs. Pitstop2 significantly decreased insoluble C9:soluble C9 ratios (lane 2 *vs* lane 4, Fig. 3b), an effect phenocopied by siRNA *vs* dynamin-2 (DNM2), the EC-specific dynamin isoform whose membrane scission activity generates endosomes (Fig. 3c). Following MAC assembly, the majority of C9 aggregates form intracellularly.

MACs vigorously assemble under cell-free conditions at pH < 6.^34,35^ While C9 is unlikely to encounter such conditions within intravascular or interstitial space, intracellular endolysosomes routinely reach intraluminal pH 4.5–5.5 to regulate vesicular maturation and tracking.^36^ Based on this, we tested whether C9 aggregates could form within the endolysosomal system. We resuspended C9 in buffers of varying pH and found that at pH ≤ 6.5, C9 began to form ring-like structures whose aggregation increased in direct proportion to buffer acidity (Fig. 3d) and protein concentration (Fig. 3e). Among MAC components, C9 showed the highest thioflavin fluorescence (Fig. 3f), altogether suggesting that endolysosomes favor C9 aggregation.

Rab5 GTPase activity regulates endolysosomal trafficking, and we asked if Rab5 activity was required for intracellular C9 aggregates. We stably transduced ECs with Rab5 DN (S43N) constructs where Rab5 is locked in an inactive GDP-bound conformation. Compared to Rab5 WT EC controls, Rab5 DN ECs lacked thioflavin staining (Fig. 3g). Rab5 activity mediates intraluminal acidification of Rab5 + vesicles, a key step required for endosome maturation,^36^ and blocking this process via buffer alkalinization with NH_4_Cl or bafilomycin, a V-ATPase inhibitor, significantly reduced thioflavin staining (Fig. 3h). In contrast, AA197–270 aggregates which bypass internalization pathways mediated by Rab5 were unaffected by these treatments (Supplementary Fig. 1c). Original and uncropped Western blot films for Fig. 3 are shown in Supplementary Fig. 4. C9 aggregates form within the endolysosomal system in a manner requiring Rab5 activity and endosome acidification.

### C9 Becomes a Substrate for Aggrephagy.

**Figure 4:**
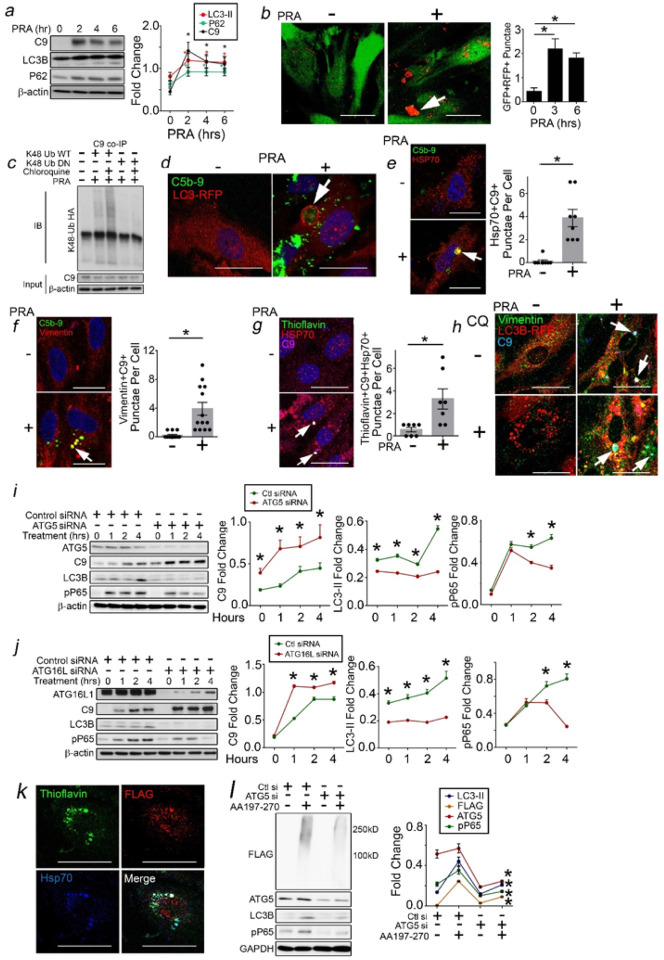
C9 Becomes a Substrate for Aggrephagy. HUVECs were treated with PRA for the times indicated prior to Western blot analysis (*a*). HUVECs transduced with mRFP-GFP-LC3B were treated with PRA and analyzed for GFP+RFP+ punctae (*b*). HUVECs were transduced with Ub WT or Ub DN and treated with PRA with or without chloroquine prior to C9 co-IPs (*c*). HUVECs transduced with LC3-RFP were treated with PRA and analyzed by I.F. (*d*). PRA-treated HUVECs were analyzed by I.F. for aggresome markers including Hsp70 (*e*), vimentin (*f*), and thioflavin (*g*). HUVECs were treated as indicated with PRA and/or pre-treated with chloroquine (CQ) for 30 minutes, and I.F. was performed 2 hours later (*h*). HUVECs were transfected with siRNA as indicated and analyzed in pulse-chase studies (*i,j*). HUVECs were transfected with AA197–270 (*k*) and analyzed by I.F. 48 hours after transfection. AA197–270 cells were co-transfected with ATG5 siRNA and analyzed by Western blot 72 hours later (*l*). Experiments repeated ≥3 times using different HUVEC donors. Scale Bar = 30mm (*b*) and 20mm (*d-h,k*) *p<0.05 using one-way ANOVA with Tukey’s post-hoc correction (*a-b,i-j,l*) or Student’s t-test (*e-g*).

Aggrephagy is a form of selective macroautophagy enabling degradation of protein aggregates which might otherwise cause cytotoxic effects within cells.^37^ Proteins containing IDRs frequently become aggrephagic substrates,^38^ prompting us to test whether C9 became a substrate for aggrephagy. PRA upregulated markers of autophagic flux including LC3-II and P62 (Fig. 4a) and generated increased GFP + RFP + LC3B punctae in ‘traffic light’ HUVECs. (Fig. 4b). In co-immunoprecipitations (co-IPs), C9 became ubiquitinylated (Fig. 4c), the first step towards targeting proteins to autophagosomes. At ~ 2 hrs, C9 became contained within LC3B + membranes (Fig. 4d) displaying markers specific for aggresomes (Fig. 4e–g). Blocking autophagosome-lysosome fusion with chloroquine (CQ) caused gross enlargement of C9 + vesicles (Fig. 4h), indicating C9 trafficking through a macroautophagic pathway. We performed pulse-chase studies in PRA-treated HUVECs and found that siRNA depletion of aggrephagy mediators, ATG5 or ATG16L, increased C9 (Fig. 4i,j). To strengthen these data, we tested AA197–270 in HUVECs. AA197–270 (FLAG) colocalized with Thioflavin + Hsp70 + aggresomes (Fig. 4k), and AA197–270 cells showed high molecular weight aggregates and increased autophagic flux which became decreased with ATG5 siRNA (Fig. 4l). Original and uncropped Western blot films for Fig. 4 are shown in Supplementary Fig. 5. These data indicated that intracellular C9 becomes an aggrephagic substrate.

### C9 Aggrephagy Activates NF-kB and Causes EC Activation.

**Figure 5:**
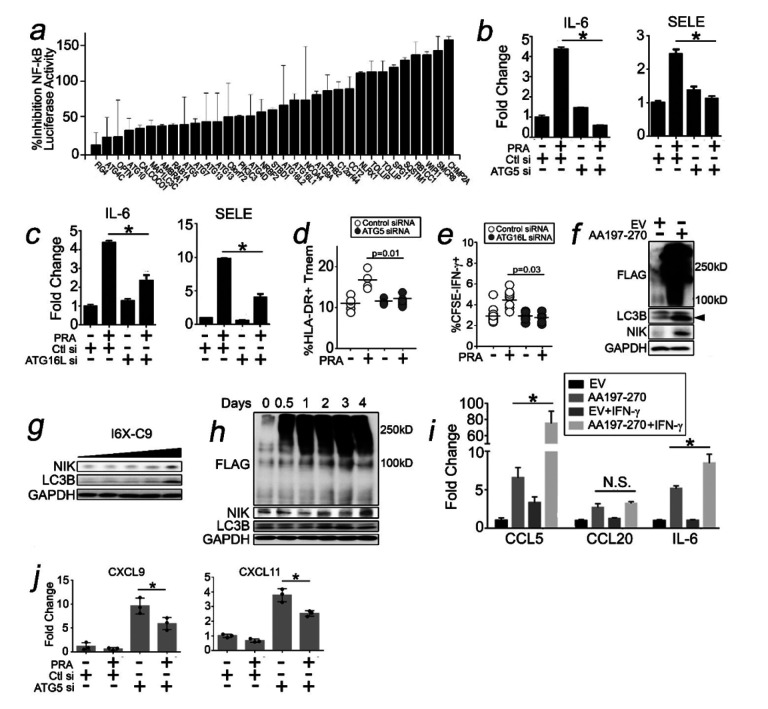
C9 Aggrephagy Activates NF-kB and Causes EC Activation. HUVECs were stably transduced with an NF-kB luciferase reporter^23^ and treated with PRA for 6 hours prior to assessing luciferase activity (*a*). HUVECs were transfected with siRNA against ATG5 (*b*) or ATG16L (*c*) and treated with PRA for 4 hours prior to performing qRT-PCR. HUVECs transfected with siRNA against ATG5 (*d*) or ATG16L (*e*) were cocultured for 10 days with alloimmune CD4+CD45RO+ T cells, and T cells were harvested for FACS analysis. HEK293 cells were transfected with AA197–270 and analyzed by Western blot as indicated (*f-h*). HEK293 cells were pre-treated with or without IFN-g (50ng/mL) for 48 hrs prior to transfection with AA197–270 and qRT-PCR (*i*). HEK293 cells were treated with IFN-g and transfected with ATG siRNA prior to qRT-PCR (*j*). Experiments repeated twice using different HUVEC donors (*a*), 2 HUVEC donors and 3 PBMC donors (*d,e*), and 2 separate transfectants (*i,j*). *p<0.05 using one-way ANOVA with Tukey’s post-hoc correction (*b-e,i-j*).

Aggresomes sequester and activate NF-κB proteins.^39,40^ We transduced HUVECs with an NF-kB luciferase reporter and performed a limited siRNA screen and found that siRNA against 33 of 52 genes annotated to ‘selective macroautophagy’ (KEGG) significantly inhibited NF-κB (Fig. 5a). Concurrent with this, a re-analysis of Western blots in Fig. 4i–j showed that siRNA against autophagy genes in PRA-treated HUVECs reduced phosphoP65 (pP65), and this concurrently reduced transcripts previously found to be NF-kB-dependent (Fig. 5b,c).^21^ Human ECs express sufficient costimulatory molecules to directly prime CD4 + CD45RO + memory T cells (Tmem) in EC:T cell cocultures,^22^ and ECs transfected with siRNA *vs* autophagy genes showed significantly reduced ability to elicit activation (HLA-DR), effector (IFN-g) and proliferative (CFSE dilution) responses in alloimmune Tmem (Fig. 5d,e).

Orthogonally, AA197–270 aggregates upregulated NF-kB marked by NIK (Fig. 5f) in both a dose- and time-dependent manner (Fig. 5g,h). To identify salient genes and/or pathways, we performed RNA seq which showed 2,160 and 2,528 genes that were significantly upregulated and downregulated, respectively, by AA197–270 aggregates (Supplementary Fig. 1d). Notably, GSEA uncovered a gene signature for autophagy (Supplementary Fig. 1e) as well as signatures related to allograft rejection, IFN-g, and NF-kB responses (Supplementary Fig. 1f). NF-kB and interferon signaling genes were strongly upregulated in AAV197–270 cells (Supplementary Fig. 1g). Based on this, we examined functional intersection between these pathways, specifically testing IFN-g priming of NF-kB genes, a phenomenon observed in PRA-treated HUVECs^22^ as well as macrophages where IFN-g may prime inflammasomes. We pre-treated AA197–270 cells with IFN-g and noted that inflammatory genes previously found to be NF-kB-dependent in PRA-treated HUVECs^22^ including CCL5 and IL-6 became synergistically or additively potentiated, respectively, by IFN-g (Fig. 5i). Similar to PRA-treated HUVECs, gene-specific knockdowns of ATG5 significantly reduced NF-kB (Fig. 4l) and inflammatory genes in AA197–270 cells (Fig. 5j). Original and uncropped Western blot films for Fig. 5 are shown in Supplementary Fig. 5. Aggrephagy of C9 promotes NF-κB and EC activation.

### ZFYVE21 is Required for C9 Aggrephagy.

While Rab5 activity was required to form C9 aggregates (Fig. 3c), prior studies have linked Rab5 activity to aggregate degradation, i.e., aggrephagy.^37^ ZFYVE21 is a conserved Rab5-associated protein implicated in cell motility,^41^ and we previously found that ZFYVE21 mediated NF-kB activity.^24,26^ The functions of ZFYVE21 have not been connected to macroautophagy, and in this context we tested roles for ZFYVE21 in mediating C9 aggrephagy.

**Figure 6:**
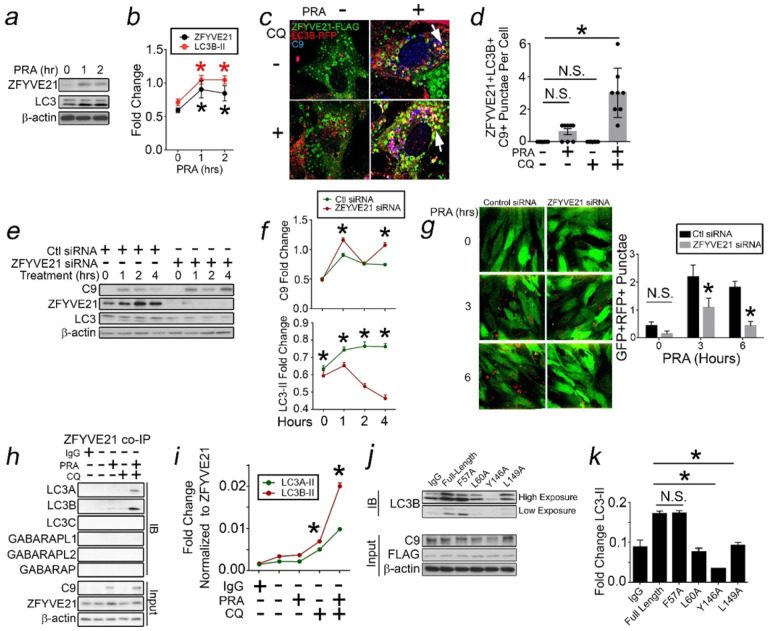
ZFYVE21 is Required for C9 Aggrephagy. HUVECs were treated with PRA at the times indicated and analyzed by Western blot (*a,b*). HUVECs transduced with ZFYVE21-FLAG and LC3B-RFP were treated with PRA containing C9-AF647 protein (10mg/mL) and either with or without CQ for 2 hours prior to analysis by I.F. (*c,d*). In pulse-chase studies, HUVECs were transfected with ZFYVE21 siRNA, treated with PRA, and soluble and insoluble fractions were analyzed by Western blot (*e,f*). HUVECs transduced with mRFP-GFP-LC3 constructs and transfected with ZFYVE21 siRNA were analyzed by I.F. (*g*). HUVECs transduced with ZFYVE21 were treated with PRA with or without CQ prior to ZFYVE21 co-IPs and Western blot analysis (*h,i*). HUVECs were transduced with FLAG-tagged ZFYVE21 constructs containing alanine substitutions at the amino acid sites indicated prior to FLAG co-IPs and Western blot analysis (*j,k*). Experiments repeated ≥3 times using different HUVEC donors. *p<0.05 using one-way ANOVA (*b,f,i,k*) or two-way ANOVA (*d*) with Tukey’s post-hoc correction or Student’s t-test (*g*).

Rab5 + endosomes sequester ZFYVE21 to shield this protein from proteasome degradation, allowing its rapid upregulation following PRA.^24,26^ became upregulated in PRA-treated HUVECs contemporaneously with LC3-II (Fig. 6a,b). To colocalize internalized MACs with ZFYVE21, we treated HUVECs with PRA spiked with AF647-labeled C9 protein, an approach forming intracellular C9 + vesicles.^24^ ZFYVE21 colocalized with C9 + vesicles containing LC3B (Fig. 6c,d, Supplementary Fig. 2a), and ZFYVE21 + C9 + punctae became grossly enlarged with CQ (Supplementary Fig. 2b). These data indicated that ZFYVE21 trafficked through an autophagic pathway. In pulse-chase studies, ZFYVE21 siRNA ablated aggrephagic flux while potentiating C9 (Fig. 6e,f), and this ablated GFP + RFP + autophagosomes (Fig. 6g). We found that in contrast to PRA, serum starvation induced autophagic flux in ECs without upregulating ZFYVE21 (Supplementary Fig. 2c,d), and ZFYVE21 siRNA in serum-starved ECs showed no effects on LC3-II (Supplementary Fig. 2c,e), indicating a principal role for ZFYVE21 in selective macroautophagy, i.e., C9 aggrephagy, but not non-selective macroautophagy.

We tested if ZFYVE21 could interact with one or more ATG8 family proteins which critically anchor signaling proteins to aggresomes.^37^ We performed pulldowns in ZFYVE21-FLAG ECs treated with CQ to block degradation of ATG8 family proteins which might otherwise become substrates for aggrephagy.^42,43^ In co-IPs, ZFYVE21 principally interacted with LC3A and LC3B (Fig. 6h,i). ATG8 family proteins including LC3B bind adapter proteins via LC3B Interacting Regions (LIRs, W/F/Y-x-x-I/L/V where x = any amino acid) and recently identified GABARAP interacting motifs (GIMs, W/F-V/I-x-V).^43^ While ZFYVE21 did not contain GIMs, we identified 2 LIRs between AA57–60 and AA146–149 (Supplementary Fig. 2f). Alanine substitutions showed that L60, Y146, and L149 mediated ZFYVE21 binding to LC3B (Fig. 6j,k). Original and uncropped Western blot films for Fig. 6 are shown in Supplementary Fig. 6. ZFYVE21 is a mediator of C9 aggrephagy, a process required for NF-kB and EC activation.

### RNF34-P62 Complexes Mediate C9 Aggrephagy.

We sought to identify E3 ubiquitin ligases utilized by ZFYVE21 to mediate C9 aggrephagy. We perform proteomic analyses of the ZFYVE21 interactome in ZFYVE21-GFP HUVECs treated with PRA for 2 hours. Three separate preparations yielded spectra for 3,347 proteins, 586 of which were uniquely upregulated by PRA (Supplementary Fig. 2g). PRA-induced proteins included Rab5A/B/C, MAC proteins (C5/6/7/8a/8g/9), and inflammasome proteins (NLRP3, casp1)^25^ which previously appeared in prior MAC + Rab5 + proteomic datasets.^24^ We additionally noted V-ATPase subunits (ATP6V1A/B2/C1/D/E1/H) and autophagy-related proteins (ATG3, ATG8G, P62), further supporting a role for vesicular acidification and ZFYVE21 within aggresomes, respectively.

We identified upregulated proteins in our proteomic datasets containing RING and HECT domains, domains known to show E3 ubiquitin ligase activity. We cross-referenced this list against proteins showing z-scores ≥ 2 in a prior genome-wide siRNA screen for NF-kB.^23^ Among proteins identified, we focused on RNF34. RNF34 is an E3 ubiquitin ligase containing a FYVE domain allowing colocalization to Rab5 + endosomes and a RING domain enabling ubiquitinylation of immune-related substrates.^44,45^ RNF34 has not been connected to macroautophagy but was previously found to directly bind ZFYVE21 in a process that enhanced its stability in HUVECs.^26^

**Figure 7:**
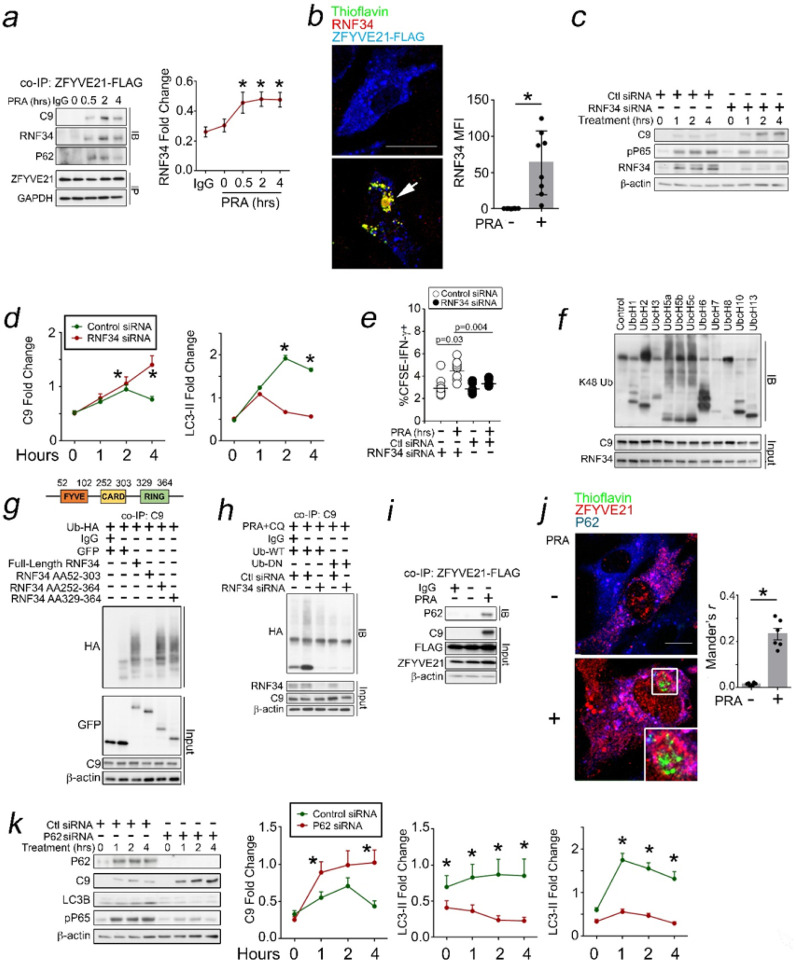
RNF34-P62 Complexes Mediate C9 Aggrephagy. HUVECs transduced with ZFYVE21-FLAG were treated with PRA at the indicated times prior to FLAG co-IP and Western blot analysis (*a*). HUVECs transduced with RNF34-HA were treated with PRA for 2 hours prior to staining for thioflavin and ZFYVE21 (*b*). HUVECs transfected with control siRNA or RN34 siRNA were analyzed in pulse-chase studies (*c,d*). HUVECs transfected with siRNA against RNF34 were cocultured with alloimmune CD4+CD45RO+ T cells, and T cells were harvested and analyzed by FACS 10 days later (*e*). Various E2 ubiquitin ligases were co-incubated with ubiquitin, E1 enzymes, RNF34 protein, and C9 protein and tested for in *in vitro* ubiquitinylation of C9 for 4 hours at 37°C (*f*). HUVECs were transfected with Ub-HA and GFP-tagged constructs encoding various regions of RNF34 prior to Ub-HA co-IPs (*g*). HUVECs were co-transfected with Ub-WT and Ub-DN along with control or RNF34 siRNA, treated with PRA, and analyzed by Western blot following C9 co-IP (*h*). PRA-treated HUVECs were transduced with ZFYVE21-FLAG prior to FLAG pulldowns and Western blot analysis (*i*). HUVECs were treated with PRA and analyzed by I.F. (*j*). HUVECs transfected with control siRNA or P62 siRNA were analyzed in pulse-chase studies (*k*). Experiments repeated ≥ 3 times using different HUVEC donors. Scale Bar = 20mm (*b*) and 10mm (*j*). *p<0.05 using one-way ANOVA with Tukey’s post-hoc correction (*a,d,e,k*) or Student’s t-test (*j*).

We thus considered whether RNF34 interacted with ZFYVE21 within aggresomes. RNF34 pulled down with ZFYVE21 following PRA treatment, and this interaction persisted to 2–4 hr, timepoints when aggresome formation occurred (Fig. 7a). At these times, RNF34 was found to heavily colocalize with ZFYVE21 + Thioflavin + aggresomes (Fig. 7b). RNF34 siRNA reduced C9 ubiquitinylation while potentiating C9 (Fig. 7c,d), and this resulted in attenuated NF-kB (Fig. 7c,d) and decreased EC-mediated T cell activation in EC:T cell cocultures (Fig. 7e). Based on this, we tested RNF34 ubiquitinylation of C9 in cell-free ubiquitinylation studies (Fig. 7f). Among E2 conjugating enzymes tested, we selected UBCH5a due to its strong activity. We found that UBCH5a allowed RNF34 to directly K48 ubiquitinylate C9 in a manner requiring its RING domain which is known to contain E3 ubiquitin ligase activity (Fig. 7g). We subsequently co-transfected CQ-treated HUVECs with RNF34 siRNA in the presence of either Ub-WT or Ub-DN constructs and found that RNF34 siRNA, like Ub-DN, blocked C9 ubiquitinylation (Fig. 7h), indicating that RNF34 ubiquitinylates C9.

Ubiquitinylated cargo require binding to proteins containing ubiquitin binding domains (UBDs).^46^ C9 and ZFYVE21 lack annotated UBDs and did not bind K48 Ub chains in the absence of E2 conjugating enzymes (Supplementary Fig. 2h). In studies above, we tested P62, a UBD-containing protein, which, in contrast to C9 and ZYVE21 showed robust binding to K48 Ub chains (Supplementary Fig. 2h). In ZFYVE21-FLAG ECs, P62 pulled down with ZFYVE21 (Fig. 7i) and heavily colocalized with ZFYVE21 + punctae concurrently showing thioflavin staining (Fig. 7j). In pulse-chase studies, P62 siRNA potentiated C9 while inhibiting LC3-II (Fig. 7k). P62 (SQSTM1) siRNA strongly inhibited PRA-induced NF-kB luciferase activity (Fig. 5a), and strongly decreased phosphoP65 in PRA-treated HUVECs (Fig. 7k). Original and uncropped Western blot films for Fig. 7 are shown in Supplementary Fig. 7. Collectively, our data support a function for ZFYVE21 as an adaptor, bridging LC3 + aggresomes to RNF34-P62 complexes to mediate C9 aggrephagy.

### Signaling Responses Related to C9 Aggrephagy Occur *In vivo*.

**Figure 8:**
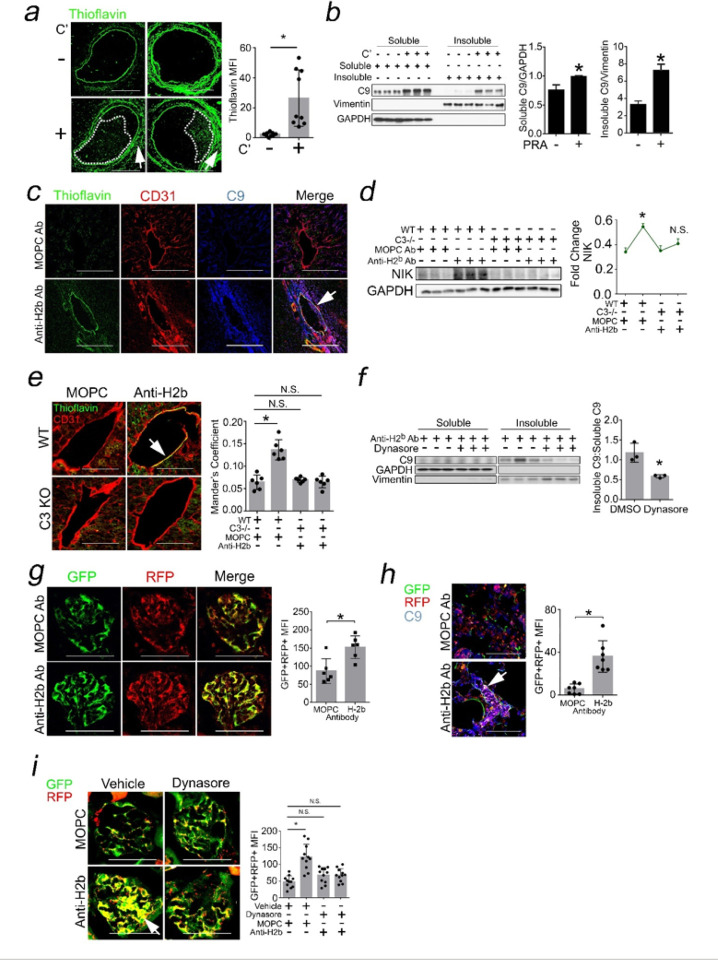
Signaling Responses Related to C9 Aggrephagy Occur *In vivo*. Human coronary artery xenografts subjected to normoxia or hypoxia and implanted into lymphoid-engrafted SCID/beige hosts were analyzed 3 weeks post-implantation (*a*). C’-treated human tissues were separated into soluble and insoluble lysate fractions and analyzed by Western blot (*b*). C57/Bl6 mice mice were injected with MOPC Ab (750mg) or anti-H2^b^ Ab (750mg), and liver tissues were harvested 24 hrs later for I.F. and kidney tissues were analyzed by Western blot (*c*, n=3 per group). C3−/− mice were injected with MOPC Ab (750mg) or anti-H2^b^ Ab (750mg), and liver tissues were harvested 24 hrs later for Western blot (*d*, n=3 per group) and I.F (*e*) analysis (n=6 per group). C57/Bl6 mice were injected with Dynasore (100mg/mouse) once daily for 2 days prior to injection with MOPC Ab or anti-H2^b^ Ab, and the soluble and insoluble fractions of kidney lysates were analyzed 24 hours later (*f*, n=3 per group). Autophagy reporter (AR) mice were injected with MOPC Ab or anti-H2^b^ Ab and glomerular ECs were analyzed for GFP+RFP+ MFIs (*g*) and GFP+RFP+C9+ MFIs (*h*, n=6 per group) 24 hrs later. AR mice were injected with Dynasore (100mg/mouse) once daily for 2 days prior to injection with MOPC Ab or anti-H2^b^ Ab, and glomeruli were analyzed 24 hours later (*i*, n=9 per group). Scale Bar = 10mm (*a,h*), 40mm (c), and 20mm (*e,g-i*). *p<0.05 using one-way ANOVA with Tukey’s post-hoc correction (*d-e,i*) or Student’s t-test (*a-b, f-h*).

We examined the relevance of salient signaling processes *in vivo*. We initially tested whether C9 aggregates could occur in AV. To do this we employed a humanized model of ischemia reperfusion injury (IRI).^27^ In this model, human artery segments are subjected to hypoxia in organ culture prior to being implanted as interposition xenografts in descending aortae of SCID/beige immunodeficient mice pre-engrafted with human lymphoid cells. This protocol activates complement (C’) and forms MACs in adluminal ECs but also the media, a region typically spared from collagen deposition in AV.^9^ This allowed us to assess thioflavin staining without confounding collagen fluorescence which complicated our prior anayses in CABMR biopsies (Fig. 1). Compared to controls, C’-treated grafts developing AV showed increased thioflavin staining in medial regions (Fig. 8a), and C’-treated tissues showed increased insoluble C9 (Fig. 8b). Our data showed that C9 aggregates may form during AV.

To further test C9 signaling responses, we employed a second approach where we injected C57/Bl6 mice (H-2^b^) with anti-H2^b^ Ab, a treatment forming non-cytolytic MACs on ECs.^26^ Following treatment with anti-H2^b^ Ab but not but not control MOPC Ab, C9 + ECs co-staining for thioflavin were visualized (Fig. 8c). Concurrent with this, anti-H2^b^ Ab-treated hosts showed increased NIK whose intensity became significantly attenuated in C3^−/−^ mice (H-2^b^) that bind IgG but lack the ability to form MACs (Fig. 8d). Concomitant with loss of NF-kB activity, C3−/− mice showed decreased thioflavin staining in ECs (Fig. 8e), indicating a role for terminal C’ activation for aggregate-induced EC activation. Insoluble C9 aggregates were significantly reduced by Dynasore (Fig. 8f), indicating that, similar to PRA-treated HUVECs, C9 aggregates formed intracellularly *in vivo*. Autophagy reporter (AR) mice are on the C57/Bl6 background (H-2^b^) and express mRFP-GFP-LC3B, allowing autophagic flux to be assessed via GFP + RFP + MFIs. We injected AR mice with anti-H2^b^ Ab and observed increased GFP + RFP + regions (Fig. 8g) colocalizing with C9 (Fig. 8h), indicating C9 aggrephagy. C9 aggrephagy was blocked by Dynasore (Fig. 8i). Internalization of terminal C’ proteins induces C9 aggregates, C9 aggrephagy, and NF-kB *in vivo*.

### ZFYVE21 Mediates RNF34-Mediated Aggrephagy and NF-kB In vivo.

**Figure 9:**
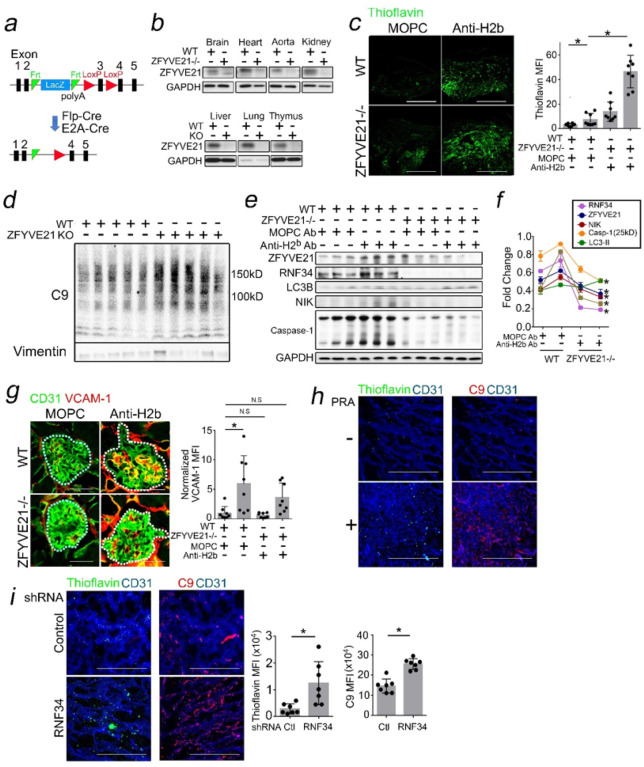
ZFYVE21 Mediates RNF34-Mediated Aggrephagy and NF-kB *In vivo*. Generation of ZFYVE21^−/−^ mice (*a,b*). Six-week-old ZFVYVE21^−/−^ mice or littermate controls (n=3 per group) were injected with MOPC Ab or anti-H2^b^ Ab and kidney tissues were analyzed for thioflavin by I.F. (*c*). WT and ZFYVE21^−/−^ mice were treated with anti-H2^b^ Ab, and 24 hours later the insoluble lysate fraction was analyzed by Western blot (*d*, n=5 per group). Kidney lysates from WT and ZFYVE21^−/−^ mice were treated as indicated for 24 hours and, total lysates were analyzed by Western blot (*e,f*, n=3 per group) or by I.F. (*g*, n=6–8 per group). HUVECs were embedded in collagen-fibronectin gels and implanted subcutaneously into flanks of SCID/beige mice. Three weeks later, mice were intravenously injected with 200mL PRA, and gels were harvested and analyzed by I.F. 24 hours later (*h*, n=7 per group). HUVECs were transduced with shRNA as indicated prior to gel embedding and implantation into SCID/beige mice. Three weeks later, mice were intravenously injected with 200mL PRA, and gels were harvested and analyzed by I.F. 24 hours later (*i*, n=6–7 per group). Scale Bar = 100mm (*c,h,i*) and 50mm (*g*). *p<0.05 using one-way ANOVA (*g*) or two-way ANOVA with Tukey’s post-hoc correction (*c*) or Student’s t-test (*h,i*).

To examine role(s) for ZFYVE21 *in vivo*, we generated ZFYVE21^fl/fl^ mice lacking exon 3 which mediates ZFYVE21 colocalization to early endosomes.^41^ Per Fig. 9a, we generated global ZFYVE21^−/−^ mice. After confirming loss of ZFYVE21 in multiple tissues (Fig. 9b), we injected ZFYVE21^−/−^ mice with anti-H2^b^ Ab and relative to age- and gender-matched littermate controls, ZFYVE21^−/−^ mice showed increased thioflavin staining ((Fig. 9c) and increased insoluble C9 (Fig. 9d). ZFYVE21^−/−^ kidneys treated with anti-H2^b^ Ab showed increased LC3B which, like P62, is known to become an autophagic substrate *in vivo*,^42,43^ indicating decreased autophagic flux (Fig. 9e). Notably, loss of ZFYVE21 virtually abolished expression of RNF34, and tissue loss of RNF34 was associated with significantly attenuated NF-kB marked by NIK (Fig. 9e,f) and reduced EC activation marked by VCAM-1 in glomerular ECs (Fig. 9g).

To further delineate roles for RNF34, we utilized a third approach incorporating collagen-bronectin gels.^24,26^ HUVECs were embedded in collagen-bronectin gels and implanted subcutaneously into SCID/beige mice, allowing HUVECs to self-assemble into perfused microvessels*in vivo*.^47^ Three weeks post-implantation, hosts bearing collagen-bronectin gels and injected with PRA showed perfused Ulex + microvessels co-staining for C9 and VCAM-1 (Supplementary Fig. 2i). PRA-treated gels additionally showed increased punctate thioflavin and C9 staining under I.F. (Supplementary Fig. 2i, Fig. 9h). In follow-up studies, HUVECs were transduced with control or RNF34 shRNA prior to gel embedding and subcutaneous implantation. Here, we found that RNF34 shRNA significantly potentiated both thioflavin staining and C9 MFIs (Fig. 9i), indicating a role of RNF34-mediated C9 aggrephagy *in vivo*. Original and uncropped Western blot films for Fig. 8 are shown in Supplementary Fig. 8. ZFYVE21 stabilizes RNF34 to mediate C9 aggrephagy, NF-kB, and EC activation *in vivo*.

### ZFYVE21 in ECs Dictates Alloimmune Tissue Injury.

**Figure 10:**
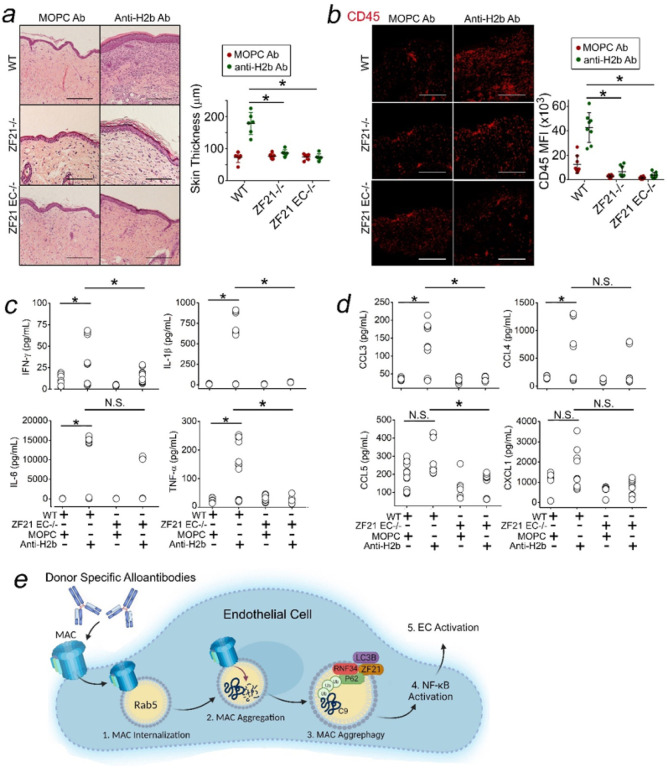
ZFYVE21 in ECs Dictates Alloimmune Tissue Injury. Male ZFYVE21^−/−^ mice, ZFYVE21 EC^−/−^ mice, and age-matched littermate controls (WT) were treated with MOPC Ab (750mg) or anti-H2^b^ Ab (750mg). Twenty-four hours later, Ab-treated skin were harvested as full-thickness skin grafts and placed onto dorsal flanks of female SCID/bg immunodeficient mice who passively received 10×10^6^ female WT C57/Bl6 splenocytes intra-peritoneally. WT, ZFYVE21^−/−^, and ZFYVE21 EC^−/−^ skin grafts were analyzed 21 days later for epidermal thickening (*a*, n=5–7 per group) and CD45+ immune cell infiltrates (*b*). Sera from hosts receiving skin grafts from littermate controls or ZFYVE21 EC^−/−^ were analyzed for cytokines (*c*) and chemokines (*d*) as indicated. Schematic showing how C9 aggregates function as intracellular alarmins (*e*). Scale Bar = 100mm (*a,b*). *p<0.05 using one-way ANOVA (*c,d*) or two-way ANOVA with Tukey’s post-hoc correction (*a,b*).

We analyzed effects of ZFYVE21 in a skin allograft model of CABMR.^26^ In CABMR, ECs become principal targets for MACs.^6,15^ To define roles for ZFYVE21 in ECs, we crossed Cdh5-Cre x ZFYVE21^fl/fl^mice to generate ZFYVE21 EC^−/−^ mice. Subsequently, male ZFYVE21 EC^−/−^ mice, ZFYVE21^−/−^ mice, and littermate controls (WT) were injected with anti-H-2^b^ Ab to form MACs, and twenty-four hours later MAC-treated male skin grafts were placed onto SCID/bg recipients passively receiving female splenocytes. Twenty-one days later, compared to MOPC Ab-treated controls, WT skin grafts treated with anti-H2^b^ Ab showed increased epidermal thickening (Fig. 10a) and CD45 + immune cell infiltrates (Fig. 10b), both of which became significantly reduced in similarly-treated ZFYVE21 EC^−/−^ and ZFYVE21^−/−^ skin grafts. We noted that the tissue readouts above were reduced in ZFYVE21 EC^−/−^ mice to a degree approximating that of ZFYVE21^−/−^ mice globally lacking ZFYVE21, indicating a principal role for ZFYVE21 in ECs in mediating alloimmune tissue injury in our disease model. On further phenotyping, we found that ZFYVE21 EC^−/−^ grafts showed decreased cytokines (Fig. 10c) and chemokines (Fig. 10d) *vs* comparably-treated WT hosts. Finally, to generalize findings we analyzed public RNA seq data from complement-mediated conditions including CABMR (n = 110), rheumatoid arthritis (n = 23), and lupus nephritis (n = 21). These analyses showed significant correlations between genes marking EC activation and aggrephagy (Supplementary Fig. 2j) as well as moderate-high correlations between aggrephagy genes with ZFYVE21 (left, Supplementary Fig. 2k) and RNF34 (right). ZFYVE21 in ECs modulates MAC-induced tissue injury *in vivo*.

## DISCUSSION

We provide data supporting the concept that MACs may disrupt proteostasis to behave as non-cytolytic, intracellular alarmins (Fig. 10e). Surface-bound MACs become internalized into Rab5 + vesicles (1) whose acidification promotes aggregates of C9 (2) that induce aggrephagy (3). In order for C9 aggrephagy to occur, ZFYVE21 acts as an adaptor, bridging LC3A/B on aggresomes to RNF34-P62 complexes. C9 aggrephagy activates NF-kB (4) and induces non-cytolytic EC activation (5), a process required for CABMR-mediated tissue injury.

Despite their initial surface distributions, MACs appear to exert the bulk of their immune effects with respect to NF-kB intracellularly. This spatially restricted feature is compatible with known alarmins including certain aggregate-prone proteins like amyloid precursor protein (APP)^48^ and prion protein (Prp)^49^ which basally localize at the cell surface and become immunogenic upon forming intracellular aggregates. In contrast to these amyloidogenic proteins as well as intracellular C’ proteins including C3^50–53^ and C5,^54,55^ C9 is introduced into intracellular space in a cell non-autonomous manner. As C9 has been detected in up to ~ 95–99% of amyloid plaques^56^ we surmise that C9 may propagate inflammation by forming de novo protein aggregates secondary to those generated by amyloidogenic proteins.

A recent study identified an endocytic pathway, termed aggregation-dependent endocytosis (ADE), allowing internalization of aggregates formed at the cell surface.^57^ We observed that endocytic blockade did not fully abrogate thioflavin staining, indicating that a residual pool of protein aggregates had formed on EC surfaces (Fig. 3a–c), a compartment where alloAbs and MACs initially assemble. While intracellular MACs significantly contributed to total levels of protein aggregates, a contribution of ADE remains untested in our current models.

## METHODS

### PRA Treatment.

‘High’ panel reactive antibody (PRA) sera were obtained as pooled, de-identified sera from the tissue typing laboratory at Yale New Haven Hospital. ‘High’ PRA sera were taken from renal transplant candidates showing allo-sensitization of ≥ 80% and negative testing for numerous infectious agents.^21^ Prior to use, PRA sera was supplemented with human complement (Sigma, #S1764) at a ratio of 1 vial of lyophilized human complement per 25mL PRA sera. PRA sera supplemented as above can be used for 3 weeks, after which complement activity deteriorates. For PRA treatment, HUVEC were pre-treated with IFN-g (50ng/mL, Invitrogen) for 48–72h prior to placement in gelatin veronal buffer (GVB, Sigma) at 25% v/v for the indicated times. In pulse-chase studies, HUVECs were pulsed with GVB containing 25% v/v PRA sera for 4h prior to washing and chased using GVB buffer alone for the indicated times. For treatment of human kidneys in organ culture, PRA was added at 1:4 ratio with GVB, for 6 hours prior to analysis.

PRA sera were fractionated into IgG- and IgG + fractions as previously described.^21^ Total IgG concentrations were first determined in intact sera prior to fractionation by ELISA (Invitrogen). Then, per manufacturer’s specifications using a MAbTrap Kit (GE Healthcare, Piscataway, NJ), 500μL of neat sera were diluted 1:1 in binding buffer and passed through the provided column pre-equilibrated with binding buffer. The column was washed with 5mL binding buffer to collect IgG- fractions, and the IgG + fraction was eluted using 5mL of elution buffer containing 650μL of neutralizing buffer. The 5mL volumes of IgG + and IgG- fractions were then serially concentrated and re-diluted in PBS using five 30 minute spins at 2100 × g in Amicon Ultra Centrifugal Filter Devices (EMD Millipore). All IgG + fractions were brought to a final concentration equivalent to the total IgG concentration prior to sera fractionation. All isolated fractions were then used at 1:10 dilution in gelatin veronal buffer.

### HUVEC Cell Culture Treatments.

All protocols were approved by the Yale IRB.

HUVECs were isolated as healthy, de-identified tissues from the Dept of Obstetrics and Gynecology at Yale New Haven Hospital as previously and plated onto microtiter wells pre-coated with 1% gelatin.^21^ HEK293 cells were commercially obtained (ATCC). HUVEC were pooled from 3 human donors and cultured in complete EBM media (Lonza) containing bullet supplements (Lonza). Where indicated, HUVEC were pre-treated with Dynasore (80μM), Pitstop2 (30μM) for 30min, or bafilomycin (100nM) for 24h in GVB prior to PRA treatment. For studies involving NH_4_Cl, HUVEC were placed in NH_4_Cl (10μM) dissolved in GVB for 24 hrs in GVB prior to PRA treatment. Following indicated treatments, to assess cellular aggregates, thioflavin T (SigmaAldrich, #T1892), was added at final concentration of 5μM to HUVECs for 30min at 37°C prior to fluorescence measurements. Thioflavin T fluorescence was measured at an excitation wavelength of 349nm and emission wavelength of 454nm using ≥ 6 experimental replicates per treatment group (Molecular Devices, SpectraMax iD3).

CD4 + CD45RA- HLA-DR- T cells were isolated from human PBMCs following depletion with anti-human CD45RA Ab (eBioscience, clone H100) and HLA-DR Ab (clone LB3.1, gift from Jack Strominger, Harvard University) using anti-human CD4 conjugated magnetic beads according to manufacturer’s specifications (Invitrogen) and co-cultured with allogeneic HUVEC pretreated with IFN-γ for 48 h and then pre-treated for an additional 6 hours with PRA sera, control sera or complement-inactivated PRA sera in round bottom 96-well tissue culture plates (BD Biosciences) at a T cell:EC ratio of 30:1 in a volume of 200μL of RPMI 1640 containing 10% fetal bovine serum with 2% L-glutamine and 1% penicillin/streptomycin. T cell proliferation assayed by labeling T cells with CFSE at 5μM (Invitrogen) and assessed by flow cytometry at seven days. All samples were acquired using a FACSCalibur flow cytometer (Becton Dickinson) and analyzed using FloJo computer software (TreeStar, Inc., Ashland, OR). Intracellular cytokine staining of activated T cells was also performed after 10–14 days of co-culture. To do so, PMA (50 ng/mL) and ionomycin (500 ng/mL, Sigma, St. Louis, MO) were added to culture medium 6 hours prior to staining and monensin (eBioscience, San Diego, CA) at 2μM was added 4 hours prior to fixation and permeabilization with Cytofix/Cytoperm staining per the manufacturer’s specifications (BD Biosciences). Permeabilized cells were stained using antibodies against IFN-γ (Biolegend, #502516) and IL-17 (eBioscience, #17-7179-42) at 1:50 dilution and analyzed using a FACSCalibur flow cytometer (BD Biosciences).

### HUVEC Lysate Treatments.

For co-immunoprecipitations, cells were lysed in RIPA buffer without SDS (Cell Signaling) containing protease inhibitor tablets (Roche, 1 tablet per 10mL RIPA buffer) in 1.5mL Eppendorf tubes with gentle agitation for 1 hr at 4°C. Following this incubation, lysates were incubated with 1–3ug of antibody against Rab5 (Santa Cruz #sc,8008), ZFYVE21(Novus Biologicals, #H00079038-B01P), HA-Tag (Bethyl,# A190–138A), Ubiquitin (Thermofisher, # 13–1600) at 4°C overnight. The next day samples were incubated with 25μL Protein A/G Magnetic Beads (Thermofisher, #88802) for 1.5 hr at room temperature and then was washed using TBS containing 3% Tween-20 three times prior to Western blotting as below. Whole cell lysates samples were harvested according to manuals of Pierce Protein A/G Magnetic Beads.

Following the above, 4X Laemli’s buffer (12μL) and 1mM DTT (6μL) were added to 32.5μL sample, and this mixture was heated for 95°C for 13min. Subsequently, samples were loaded onto pre-cast polyacrylamide gels (Bio-Rad), and proteins were electrophoretically separated and transferred to methanol-activated PVDF membranes at 4°C for 90 minutes. Membranes were washed for 15 minutes three times using Tris-buffered saline containing 0.1% Tween-20 pH 7.4 (TBS-T, AmericanBio), blocked with TBST containing 3% bovine serum albumin (Sigma) for 1 hr at room temperature, and incubated with primary antibody at 4°C overnight. Antibodies used for Western blotting were all used at 1:1000 dilution and included ZFYVE21 (Biorbyt, # orb221973), RNF34 (invitrogen, # PA5–113296), Rab5 (Santa Cruz Biotechnology, #sc-46692), K48-conjugated ubiquitin (Cell Signaling, #8081), caspase-1 (Santa Cruz Biotechnology, #sc-392736), SQSTM1/p62(Cell Signaling, #5114s), C9 (Abcam, # ab173302), ATG5 (Cell Signaling, # 12994s), ATG16L1 (Cell Signaling, # 8089s), LC3B (Cell Signaling, # 83506s), Flag Rabbit (Cell Signaling, # 2368s), Flag Mouse (Sigma, # F1804), RFP (Abcam, # ab62341), GFP Goat (Rockland, # 600101215) and ß-actin (Cell Signaling, # 4970S).

HUVECs were fractionated into soluble and insoluble fractions as previously described.^64^ For proteinase K treatments, soluble and insoluble C9 fractions were incubated with 12.5 μg/ml proteinase K for 30 min at RT prior to Western blot analysis.

### Immunofluorescence Staining.

For immunostaining, HUVEC were grown on glass coverslips, fixed and permeabilized with ice cold methanol for 15min, blocked with PBS containing 0.1% Tween and 5% FBS for 1hr at room temperature (PBST). Primary antibodies were then incubated overnight at 4C at 1:200 dilution using the following antibodies: Ulex (Vector Labs, #B-1065), p-P65 (Santa Cruz, #sc-8008), ZFYVE21 (Atlas, # HPA055721), vimentin (Invitrogen, # MA1–19168), HSP70 (Invitrogen, # MA3–007), HA-Tag Rabbit (Cell Signaling, # 3724S),HA-Tag Rabbit (Cell Signaling, # 3724s), HA-Tag Mouse (Cell Signaling, # 2367s), Flag Rabbit(Cell Signaling, # 2368s), Flag Mouse(Sigma, # F1804). For thioflavin staining, slides were incubated with 5μM thioflavin T (Sigma, #596200) dissolved in PBS. The next morning slides were washed 3 times in PBS and incubated 2 hours at room temperature with secondary antibodies (Invitrogen, #A-31571, #A-31573, #A-31572, #A-21202, #A-11055, #A-21206, # A-31570, # A21447, # A-21432) at 1:200 dilution. Following staining, slides were washed, air dried, and cover slipped using a DAPI mounting media (ImmunoGold with DAPI, Invitrogen). Immunofluorescence was visualized using a Leica SP8 confocal microscope.

For I.F. analyses of FFPE patient biopsies, sections were deparaffinized and hydrated, TUNEL staining were conducted according to the manufacturer’s specifications (Invitrogen, #C10619). Antigen retrieval was performed at 95°C for 1 h in Antigen Unmasking Solution (VectorLabs) and stained using antibodies against ZFYVE21 (Atlas), C5b-9 (Dako, # M0777) at 1:200 dilution at 4°C overnight prior to addition of secondary Abs (1:500) and thioflavin T 5μM for 2 h at room temperature.

### siRNA Transfection of HUVECs.

HUVEC were pre-treated with IFN-g for 48 hours prior to siRNA transfection. siRNA targeting ZFYVE21, ATG5, ATG16L1, DMN2 and RNF34 or non-targeting siRNA (target sequence UAA CGA CGC GAC GUA A) were purchased as pooled siRNA (Horizon Discovery) and transfected into HUVEC at ~ 50–70% confluency in 24-well plates (BD Falcon). siRNAs were diluted at 20–40nM concentration in Opti-Mem culture media (Gibco) and mixed at equal volume with RNAiMax transfection reagent (Invitrogen) diluted 1:50 in Opti-Mem for 15 minutes at room temperature as per the manufacturer’s specifications. This mixture was then added to HUVEC cultures at 1:6 ratio for 37°C for 6 hours prior to washing and buffer exchange with EGM2. IFN-g was added at 50ng/mL, and cells were then analyzed by Western blot, luciferase assay, RT-PCR, or T cell functional assays 48 hours later (72 hrs after transfection).

### Real Time Quantitative Reverse Transcription-Polymerase Chain Reaction (Quantitative RT-PCR.

RNA was isolated from treated HUVEC according to the manufacturer’s specifications (Qiagen) and reverse transcribed (Applied Biosystems, Foster City, CA). Respective cDNA was amplified in a CFX Realtime System (Biorad, Hercules, CA) at a volume of 20μL containing dilutions of 1:20 Taqman probe (Applied Biosystems), 1:2 Taqman Gene Expression Master Mix (Applied Biosystems), and 1:10 cDNA in ddH-2O. RT-PCR gene probes were purchased from Applied Biosystems [IL6 (#Hs00985639_m1), SELE (#Hs00950401_m1)]. For amplification, samples were heated to 50°C for 2 minutes for once cycle, 95°C for 10 minutes for one cycle, and then 40 cycles where samples were heated to 95°C for 15 seconds proceeded by 60°C for 1 minute.

### Cell-Free Studies.

In cell-free experiments involving complement proteins, human complement C9 (Complement Technology, #A126) was incubated at 0.125μg/μL in 50mM NaCl titrated to the pH indicated in the text and containing thioflavin (5μM) at a final volume of 50μL/well and gently rocked at 37°C for the times indicated prior to assessing fluorescence at excitation wavelength at 349nm and emission wavelength at 454nm using ≥ 6 experimental replicates per treatment group. In other studies, human complement proteins (C5 #A120, C6 #A123, C7, #A124, C8, #125, and C9, #A126) were incubated at 0.2μg/μL in a hypotonic buffer (50mM NaCl) titrated to pH 4.5 as indicated in the text. In cell-free experiments involving ubiquitin (Ub), human ZFYVE21 (0.4 μg), C9 (0.4 μg), P62 (0.4 μg), and Ub (1.0 μg) were co-incubated at a final volume of 50μL with gentle rocking at 4°C overnight prior to performing co-immunoprecipitations using an anti-Ub mAb (Invitrogen, #13–1600).

### Molecular Cloning Studies.

NF-kB luciferase lentiviral particles were commercially obtained (Cignal Reporter Assay, Qiagen) and used at an MOI of 20 to infect HUVECs for 8 hrs two times as previously described.^23^ The following reporter constructs were obtained from Addgene: mCherry-Rab5 WT (a gift from Gia Voeltz, plasmid #49201). mCherry-Rab5 DN (a gift from Sergio Grinstein, plasmid #35139), pRK5-HA-Ubiquitin-K48R (Addgene plasmid # 17604), pRK5-HA-Ubiquitin-WT (Addgene plasmid # 17608), HA-p62 (Addgene plasmid #28027), pcDNA3-GFP-LC3-RFP-LC3ΔG (Addgene plasmid, #168997), pmRFP-LC3(Addgene plasmid #21075).

### EC:T Cell Cocultures.

All protocols were approved by the Yale Institutional Review Board (#0601000969). PBMCs were isolated from leukopacks using density centrifugation as described previously and cryopreserved in liquid nitrogen.^21^ CD4 + CD45RO + T cells were isolated from thawed cryovials using magnetic bead separation kits (Miltenyi) with HLA-DR Ab (clone L243, Novus #NB100-77855) and CD45RA Ab negative depletion (10μL per cryovial, eBiosciences, 14-0458-82).For EC:T cell cocultures, HUVEC isolated from a single donor were grown in U-bottom 96-well microtiter plates, pretreated with human IFN-g (50ng/mL, Invitrogen) for 48–72hr. On the day of the experiment, ECs were placed in gelatin veronal buffer containing 25% v/v of PRA sera for 6 hr prior to addition of human CD4 + CD45RO + T cells which were added at 0.5–1×106 cells/well at a volume of 200μL in RPMI (Gibco) supplemented with 5% FBS, 1.5% L-glutamine, and 1% penicillin/streptomycin. T cells were harvested 10–14 days after co-culture in a humidified incubator at 5% CO2 and 37°C prior to FACS analysis.

### Mouse Studies.

All protocols were approved by the Yale IACUC (#2023–20175). Human artery grafts were obtained from consenting heart transplant donors at Yale New Haven Hospital. Donors were obtained from patients with non-ischemic cardiomyopathy (NICM) angiographically lacking pre-existing stenotic lesions. Human artery segments approximating the diameter of murine descending aortae were dissected and placed into organ culture in DMEM with 5% FBS at 0% FiO2 5% CO2 for 37°C for 8 hours prior to surgical reimplantation as interposition grafts into descending aortae of recipient female 6–12-week old SCID/beige mice pre-engrafted with human lymphoid cells as previously described.^27^ Grafts were harvested three weeks later for I.F. analysis.

Adult mice aged 6–12 weeks old were used in our studies. Mice were housed in a standard animal maintenance facility under a 12-hour light/12-hour dark cycle. Wild-type C57/Bl6 mice (Jackson Labs, #000664), autophagy reporter mice (Jackson Labs, #027139), and C3^−/−^ mice (#029661, Jackson Labs) were injected i.v. via tail vein injection with 750μg anti-mouse MOPC Ab (Ichor, clone MPC-11) or 750μg anti-H-2^b^ Ab (Ichor, clone 8-24-3), and twenty-four hours later, kidneys were harvested for I.F. analyses and co-IP studies as indicated.

To generate global and EC-specific ZFYVE21^−/−^ mice, C57BL/6N-A^*tm1Brd*^

*Zfyve21*^*tm2a(EUCOMM)Wtsi/BayMmucd*^ mice were initially purchased from Mutant Mouse Resource & Research Centers (catalog 043932-UCD). To generate this strain, the L1L2_Bact_P cassette was inserted at position 111823434 of chromosome 12 upstream of exon 3, a region we found was required for ZFYVE21 localization to early endosomes. Founder mice were crossed with B6N.129S4-Gt(ROSA)26Sortm1(FLP1)Dym/J (#016226, Jackson Labs) to generate Zfyve21^fl/fl^mice which were then crossed to either Ella-Cre mice (#003724, Jackson Labs) or Cdh5-Cre mice (#006137, Jackson Labs) to obtain global and EC-specific ZFYVE21^−/−^ mice, respectively, both of which express the H-2^b^ haplotype.

For skin graft experiments, skin from 6–12 week-old male C57/Bl6 mice, ZFYVE21-deficient strains, and littermate controls were treated with MOPC Ab or anti-H-2^b^ Ab as above, dorsal skin segments were harvested 24 hrs after Ab injection, and implanted on the dorsal flanks of female SCID/bg hosts (#CBSCBG-F, Taconic) as full-thickness xenografts. Seven days later, mice were injected i.p. with 5×10^6^ female C57/Bl6 splenocytes, and skin grafts were harvested three weeks later for analysis. Epidermal thickness was quantified in 3–5 hpfs using morphometry, and mononuclear cell infiltration was estimated by 3 blinded observers.

### Proteomic Analyses.

For proteomic analyses, HUVECs from 3 separate donors were grown to confluence in 15 T175 flasks prior to being treated with vehicle (gelatin veronal buffer) or PRA 25% v/v for 45 minutes. Subsequently, cells were harvested and GFP pulldowns were performed according to the manufacturer’s specifications using GFP-Trap agarose beads (#gta, ProteinTech). Proteins were eluted from agarose beads and subjected to trypsin digestion and label-free proteomic analysis by mass spectrometry-liquid chromatography (MS-LC) as previously described.^24^

### Multiplex Laser Bead Assay.

Polystyrene beads containing fluorescent dyes were coated with capture antibody specific for a given protein analyte. Color-coded beads were then analyzed using a bead analyzer (Bio-Plex 200) containing a dual-laser system where the fluorescent dye within each bead is activated, and a second laser excites the fluorescent conjugate (streptavidin-PE) that has been bound to the beads during the assay. The amount of conjugate detected by the analyzer is in direct proportion to the amount of the target analyte which can be quantified using a standard curve (Eve Technologies).

### Statistical Methods.

Paired analyses were performed using two-tailed Student’s t test and multiple comparisons were performed using a one-way or two-way ANOVA followed by Tukey’s pairwise comparison test using Origin computer software. p-values < 0.05 were considered statistically significant. Standard deviations are reported throughout the text.

## Data Availability

All data and methods are available from the authors upon reasonable request. The following transciptomic datasets were retrieved from the Gene Expression Omnibus: GSE147089 (n = 224) [https://www.ncbi.nlm.nih.gov/geo/query/acc.cgi?acc=GSE147089], GSE112943 (n = 21) [https://www.ncbi.nlm.nih.gov/geo/query/acc.cgi?acc=GSE112943], and GSE97779 (n = 23) [https://www.ncbi.nlm.nih.gov/geo/query/acc.cgi?acc=GSE97779]. An ‘Aggrephagy Index’ was calculated by averaging probe counts for 7 genes annotated to aggrephagy (ATG5, ATG16L, SQSTM1, ATG12, MAP1LC3B, BECN1, WDFY3), and an ‘EC Activation’ index was calculated by averaging probe counts for 3 adhesion molecules highly expressed in activated ECs (VCAM1, ICAM1, SELE).
